# Single Dose Comparative Bioavailability Study of Lisdexamfetamine Dimesylate as Oral Solution Versus Reference Hard Capsules in Healthy Volunteers

**DOI:** 10.3389/fphar.2022.881198

**Published:** 2022-04-05

**Authors:** Simona Rizea-Savu, Simona Nicoleta Duna, Dimitrios Panagiotopoulos, Roxana Colette Sandulovici

**Affiliations:** ^1^ 3S-Pharmacological Consultation & Research GmbH, Harpstedt, Germany; ^2^ Faculty of Pharmacy, Titu Maiorescu University, Bucharest, Romania; ^3^ 3S-Pharmacological Consultation & Res. SRL, Bucharest, Romania; ^4^ Labomed Pharmaceutical Company SA, Koropi, Greece

**Keywords:** lisdexamfetamine dimesylate, dexamfetamine, pharmacokinetics, bioequivalence, analytical method

## Abstract

Lisdexamfetamine is an inactive prodrug of dexamfetamine that is used for the second-line treatment of attention-deficit/hyperactivity disorder (ADHD) and moderate to severe binge eating disorder (BED). Once in the blood, the prodrug is hydrolyzed in erythrocyte cytosol, thus releasing the active dexamfetamine. We here present a fully validated HPLC-MS/MS analytical method for simultaneous determination of lisdexamfetamine and dexamfetamine in human plasma and the first published comparative bioavailability study of lisdexamfetamine including a GMP finished product formulated as oral solution. The Test (T)/Reference (R) ratios for the geometric means (%) of the primary pharmacokinetic (PK) parameters and their corresponding two-sided 90% confidence intervals (CIs) were contained within the predefined regulatory limits of 80.00–125.00% for both lisdexamfetamine and dexamfetamine. While for the lisdexamfetamine prodrug, PK results for the two formulations were slightly different due to the distinct dissolution state at administration, the PK parameters calculated for dexamfetamine were almost identical. A potential explanation of this phenomenon, already described in literature, is that biotransformation of lisdexamfetamine by red blood cells (rather than its release within the gastrointestinal tract) is the process controlling the rate of dexamfetamine delivery.

## Introduction

Lisdexamfetamine is an inactive prodrug of dexamfetamine, used for the second-line treatment of attention-deficit/hyperactivity disorder (ADHD) in children above the age of 6 years and up to adulthood, when response to previous methylphenidate treatment is considered clinically inadequate (Krishnan et al., 2008; Hutson et al., 2014). In early 2015, the United States Food and Drug Administration (FDA) also approved lisdexamfetamine dimesylate for the treatment of moderate to severe binge eating disorder (BED) in adults (Griffiths et al., 2019).

Following oral administration, lisdexamfetamine is rapidly taken up from the small intestine by active carrier-mediated transport, probably *via* peptide transporter 1 (Sharman and Pennick, 2014). Once in the blood, the prodrug is hydrolyzed in erythrocyte cytosol by an unknown aminopeptidase, thus releasing L-lysine (a naturally occurring essential amino acid) and the active dexamfetamine, with an estimated conversion efficacy of 98% (Pennick, 2010; Sharman and Pennick, 2014).

The dexamfetamine generated crosses the blood-brain barrier to access binding sites in the central nervous system and to exert therapeutic effects by increasing noradrenergic and dopaminergic neurotransmission (Ermer et al., 2010).

After administration of lisdexamfetamine dimesylate in 50 mg incremental single doses ranging from 50 to 250 mg, it was found that the pharmacokinetic parameters of dexamfetamine are dose-proportional and predictable over a wide range of prodrug doses, with low intersubject and intrasubject variability (<20%) of doses up to 150 mg (Ermer et al., 2010).

In contrast to other long-acting psychostimulant formulations in which the extended activity is dependent on controlled medication release within the gastrointestinal tract, in the case of lisdexamfetamine, biotransformation by red blood cells is the process controlling the rate of dexamfetamine delivery (Ermer et al., 2016). This characteristic makes lisdexamfetamine a very good candidate for reformulation as an oral solution, in view of increasing its acceptability in children suffering from ADHD.

While this hypothesis has been tested before using the dissolved content of lisdexamfetamine capsules (Krishnan et al., 2008), this is the first published comparative bioavailability study of lisdexamfetamine including a GMP finished product formulated as oral solution (performance not affected by excipients of a dissolved solid oral dosage form).

## Materials and Methods

### Standards and Reagents

The analytical-grade reference standards dexamfetamine hydrochloride and lisdexamfetamine dimesylate were obtained from Toronto Research Chemicals (Ontario, Canada). The analytical-grade internal standards (IS) were obtained from: Cerilliant Analytical Reference Standards (Round Rock, Texas, United States) in the case of (±)-amfetamine-d8 and Supelco Inc. (Merck subsidiary in Bellefonte, Pennsylvania, United States) in the case of lisdexamfetamine-d4. Ammonium acetate, formic acid, acetonitrile, dimethylsulfoxide, and methanol were of high-performance liquid chromatography (HPLC) grade, purchased from Merck (Darmstadt, Germany). Water was purified using Milli-Q water purification system from Millipore.

### Equipment

For the preparation of samples, two Hamilton Microlab STARLet automated robotic liquid handling units (Hamilton Company, Reno, Nevada, United States) were used, operated by Hamilton Venus 3 software. Shimadzu HPLC systems, consisting of CTC autosamplers, LC-20AD binary pumps, DGU-20A5 degassing units, and CTO-20A thermostatted columns heaters (Shimadzu, Kyoto, Japan), were used for the validation tests as well as real samples analysis. The mass spectrometers utilized for this work were an API 5500 triple-quadrupole mass spectrometer equipped with atmospheric pressure electrospray ionization interface (Turbo Spray) and an API 6500 QTRAP equipped with atmospheric pressure electrospray ionization interface (model Turbo-Spray ion Drive), both manufactured by AB Sciex (Foster City, California, United States). Analytical sequences were run in parallel on the two mass spectrometers. Study data were collected using Analyst® (Version 1.7 Applied Biosystems). MPX Driver (using MPX SW version 2.0) software was used to control the LC parameters. The software MPX-2 controlled all functions of the multiplexing, keeping a clear audit-trail of the operations, sample by sample. Each analytical sequence, generally composed by calibration curve, quality controls, and study samples, was injected in a single column, to avoid the risk of differences in quantitation between the two columns, and two sequences were run in parallel.

### Liquid Chromatography and Mass Spectrometric Conditions

A single analytical method for simultaneous quantification of dexamfetamine and lisdexamfetamine was developed, validated, and used for real samples analysis. Chromatographic separations were carried out using Ascentis Express 90A, RP-Amide (15 cm × 2.1 mm; 2.7 µm) silica packing reversed phase analytical columns. HPLC separations were carried out using a composition gradient of ammonium acetate 10 mM in water spiked with formic acid (mobile phase A) and acetonitrile (mobile phase B). Samples of 40 µl were loaded onto the column (5 µl loop injected), separated and eluted in gradient conditions. The total LC method run time was 6.5 min, with a data acquisition window of 2.5 min. Temperature of the autosamplers was maintained at 10°C nominal. The mass spectrometers were run in positive ions mode using multiple-reaction monitoring (MRM) to monitor the mass transitions. Research grade nitrogen was used as curtain gas and collision gas (CAD) while auxiliary and nebulizer were supplied with zero grade air. The Turbo Spray gas was warmed at 500°C. The resolutions for both Q1 and Q3 were set at unit. A summary of the ion transitions, declustering potentials, collision energies, and collision cell exit potentials are presented in Table 1.

**TABLE 1:** Optimal positive ions mass spectrometric conditions for multiple reaction monitoring.

Mass spectrometre	Analyte	Ion transition	Dwell time (ms)	Declustering potential (V)	Collision energy (V)	Collision cell exit potential (V)
API 5500	Dexamfetamine	136.040 >> 119.000	100	100	11	6
	Lisdexamfetamine	264.170 >> 83.900	100	100	33	14
	Amfetamine-d8 (IS)	144.094 >> 127.100	100	100	11	16
	Lisdexamfetamine-d4 (IS)	268.210 >> 87.900	100	100	21	14
API 6500	Dexamfetamine	136.040 >> 119.000	100	100	11	12
	Lisdexamfetamine	264.170 >> 83.900	100	100	27	10
	Amfetamine-d8 (IS)	144.094 >> 127.100	100	100	13	8
	Lisdexamfetamine-d4 (IS)	268.210 >> 87.900	100	100	21	14

### Calibration Curves and Quality Control Samples

Stock solutions of dexamfetamine free base and lisdexamfetamine free base in dimethylsulfoxide were prepared at a concentration of 1.000 mg/ml. These solutions were stored at −20°C. A series of working solutions for preparation of the eight points calibration curves and the plasma QC samples were obtained by mixing and diluting the stock solutions with pooled human plasma deriving from blank blood samples collected on K_2_EDTA from healthy volunteers. Spiked calibration standards were prepared with dexamfetamine and lisdexamfetamine together, at the following concentrations: 2.000/1.000–4.000/2.000–8.000/4.000–16.000/8.000–32.000/16.000–64.000/32.000–128.000/64.000–200.000/100.000 ng/ml (eight points calibration curve). Spiked QC samples were prepared with dexamfetamine and lisdexamfetamine together, at the following concentrations: 6.000/3.000–40.000/20.000–80.000/40.000–160.000/80.000 ng/ml.

### Bioequivalence and Palatability Study in Healthy Volunteers

The validated method was used for analysis of real samples from an open label, two-period, two-sequence, cross-over, randomized, single dose bioequivalence study of Lisdexamfetamine dimesylate 10 mg/ml oral solution (test formulation developed by Labomed Pharmaceutical Company SA, Greece) vs. equal dose of Elvanse 70 mg hard capsule (reference formulation, of Shire Pharmaceuticals Ireland Limited, Ireland), administered in fasting conditions. The reference product was sourced from the EU market (Germany). The test oral solution (finished dosage form, no reconstitution required) was developed and manufactured in GMP conditions in a certified facility.

The study included 32 fasting healthy volunteers (13 males and 19 females), Caucasian, adults (between 18 and 45 years of age) with a body mass index within 18.5–30.0 kg/m^2^. Upper age limit was selected based on the rationale that lisdexamfetamine dimesylate pharmacokinetics do not show age dependent features and thus, bioequivalence data collected in young adults can be considered as representative for the target pediatric or adult population (the drug being mainly intended for use in children with ADHD, with treatment into adulthood being recommended in some specific cases).

All subjects gave their written informed consent before they underwent any study-related procedures and were free to withdraw from the trial at any time. All subjects met the inclusion criteria and none of the exclusion criteria described in the protocol and were considered healthy according to the judgment of the clinical investigator based on their medical history, physical examination, 12-lead electrocardiogram (ECG), vital signs measurement, and clinical laboratory tests (hematology, clinical chemistry, and virology). COVID-19 testing was performed at screening and before each study period. All of the female subjects enrolled and dosed were non-pregnant, non-lactating, and using a highly effective method of contraception.

The study medication administration consisted of one single 70 mg hard capsule of lisdexamfetamine dimesylate (trade name: Elvanse®) or a volume of 7 ml Lisdexamfetamine dimesylate 10 mg/ml oral solution (volumetry ensured by syringe-dosing and double checked through weight measurement) per study period. Both study treatments were administered after a minimum 10 h of overnight fasting. Subjects were randomly assigned to the Test-Reference or Reference-Test treatment sequences and there was a wash-out period of 14 days between administrations. The Reference hard capsule was administered with 150 ml of still bottled water, while the Test solution administration was followed by intake of 143 ml still bottled water used for syringe and mouth rinsing.

The 28 study subjects judged by the Investigator as being taste- and smell-sensitive (based on their ability to recognize the full range of tastes and odors included in the organoleptic screening) were included in a palatability evaluation subpopulation. The palatability evaluation conducted for the Test oral solution was based on five organoleptic parameters (acceptability, bitterness, sweetness, aftertaste, and flavor), each assessed on a 5-point scale.

For the analytical determination of dexamfetamine and lisdexamfetamine plasma levels, venous blood samples of 5 ml were drawn in tubes containing K_2_EDTA as anticoagulant before study drug administration and at 0.08 (5 min), 0.17 (10 min), 0.33 (20 min), 0.50 (30 min), 0.75 (45 min), 1.00, 1.25 (1 h 15 min), 1.5 (1 h 30 min), 1.75 (1 h 45 min), 2.00, 2.33 (2 h 20 min), 2.67 (2 h 40 min), 3.00, 3.5 (3 h 30 min), 4.00, 4.5 (4 h 30 min), 5.00, 6.00, 8.00, 12.00, 16.00, 24.00, 48.00, and 72.00 h post dose.

Subjects were hospitalized, under continuous medical observation starting with 12 h before dosing and up to 48 h post-dosing in each study period. The screening and study exit examinations as well as the blood sampling visit at 72 h post dose were conducted in ambulatory conditions.

The pharmacokinetic parameters calculated for each analyte were AUC_0-t_, C_max_, T_max_, AUC_0-∞_, t_1/2,_ and MRT.

The study was conducted at the Phase I-IV Clinical Centre of 3S-Pharmacological Consultation and Res. SRL in Romania, following unconditional approval from the National Bioethics Committee for Medicines and Medical Devices and the Romanian Medicines and Medical Devices Agency. Clinical investigations were conducted according to the Declaration of Helsinki principles and Good Clinical Practice.

### Handling of Study Samples

After collection, the blood samples were centrifuged under refrigeration (10 min at 1500 (±5) g and a nominal temperature of 4°C). Plasma was separated, divided into duplicate aliquots and, within 60 min from collection, frozen for storage at −20°C nominal until shipped to the analytical laboratory. Plasma samples (first aliquot) were sent from the clinical site to the analytical facility in a thermo-insulated box containing an adequate amount of dry ice. During transport, an electronic logger was used for monitoring plasma samples temperature. Once received at the analytical laboratory, the samples were stored at −20 °C nominal until submitted to analysis. Before analysis, plasma samples were thawed, mixed for 3 min and centrifuged for 3 min at 4000 rpm and 20°C nominal. Aliquots of samples were spiked with internal standards solution mix [(±)-amphetamine-d8 and lisdexamfetamine-d4 in methanol], diluted with acetonitrile, spiked with formic acid, vortexed, and centrifuged. Supernatants were diluted with water, mixed, and centrifuged; finally, the samples have been transferred to the autosampler to be injected.

The analytical work was performed according to GLP principles, FDA requirements (FDA Bioanalytical Method Validation Guidance, 2018) and EMA requirements (EMA Guideline on bioanalytical method validation, EMEA/CHMP/EWP/192217/2009 Rev. 1 Corr. 2**, 2011). The analytical method was fully validated before starting the analysis of study plasma samples. The method was verified for linearity, quantification limits, assay specificity, between-run and within-run precision and accuracy, analyte recovery, and stability in stock solution and biological matrix under processing conditions during the entire period of storage.

### Pharmacokinetic and Statistical Analysis

Non-compartmental PK analysis was performed using SAS® statistical software, version 9.4 (SAS Institute Inc., United States). Maximum plasma concentration (C_max_) and time to reach maximum plasma concentration (T_max_) were obtained directly from the plasma values. The linear trapezoidal rule was used to calculate the area under the concentration-time curve from time zero to the last quantifiable concentration above LLOQ (AUC_0–t_). The apparent elimination rate constant (K_el_) was estimated by regression of the terminal ln-linear portion of the plasma concentration–time profile; apparent terminal half-life (*t½*) was calculated as the quotient of ln (2) and K_el_. Area under the curve to infinity (AUC_0–∞_) was estimated as the sum of AUC_0–t_ and the extrapolated area given by the quotient of the last quantifiable plasma concentration and K_el_. ANOVA was performed on ln-transformed C_max_, AUC_0-t_, and AUC_0–∞_ using the General Linear Models (GLM) procedure fitted in SAS® software using the method of least squares. The confidence interval for the ratio of the population means was calculated considering a classic (shortest) 90% confidence interval. The bioequivalence acceptance range was set to 80.00–125.00% for lisdexamfetamine C_max_ and AUC_0–t_ (primary PK parameters). The effect of sequence, period, subject within sequence and treatment on lisdexamfetamine C_max_, AUC_0-t_, and AUC_0–∞_ has been separately evaluated on ln-transformed data. The intra-subject variability (ISCV) of C_max_, AUC_0-t_, and AUC_0–∞_ was also separately determined. Lisdexamfetamine AUC_0–∞_ data was regarded as supporting evidence (secondary PK parameter). T_max_ data have been compared using the non-parametric Wilcoxon Signed-Rank Test, applied to untransformed data. The limit of statistical significance was considered *p* < 0.05. The same statistical approach described above for lisdexamfetamine was applied also for the analysis of dexamfetamine PK data, which was considered supportive information based on EMA Guidelines. Descriptive statistics were performed for all pharmacokinetic parameters.

## Results

### Method Validation and Incurred Study Samples Re-Assay Results

#### Selectivity

Analyses were performed on 10 blank plasma samples collected on K_2_EDTA from different healthy volunteers (including two lipemic and two hemolytic samples) without any addition and then with addition of internal standards mix or lisdexamfetamine or dexamfetamine or possible co-medication (acetylsalicylic acid, salicylic acid, domperidone, ibuprofen, loperamide, metamizole, and metamizole main metabolites, metoprolol, paracetamol, caffeine, metoclopramide, and theobromine at 10.000 μg/ml, each.); no peak interfering with those of the analytes or the internal standards appeared in the blank samples. Same test was applied to LLOQ samples. None of the samples showed any obvious interference.

#### Calibration Curve Fitting, Precision and Accuracy

The precision and the accuracy, at all concentrations, were satisfactory (mean within-run precision ranged from 2.16 to 5.66% for lisdexamfetamine QCs and 2.04–7.93% for dexamfetamine QCs; mean within-run accuracy ranged from 95.63 to 103.13% for lisdexamfetamine QCs and 100.44–107.58% for dexamfetamine QCs; mean between-run precision ranged from 2.56 to 4.99% for lisdexamfetamine QCs and 1.92–7.61% for dexamfetamine QCs; mean between-run accuracy ranged from 96.00 to 103.04% for lisdexamfetamine QCs and 102.07–104.50% for dexamfetamine QCs). The curves fitting was also optimal in the whole range with correlation coefficients (r) = 0.99960 for both dexamfetamine and lisdexamfetamine.

#### Extraction Recovery

The extraction recoveries of QC samples, calculated on the peak areas of lisdexamfetamine (mean recovery across the three QC levels tested: 108%), IS lisdexamfetamine-d4 (mean recovery across the three QC levels tested: 94%), dexamfetamine (mean recovery across the three QC levels tested: 86%) and IS (±)-amfetamine-d8 (mean recovery across the three QC levels tested: 93%) put in evidence that the extraction was effective at all tested concentrations with all compounds, being above 80.0% for all individual tests; therefore, adequate for an analytical method.

#### Matrix Effect

The matrix effect was also evaluated. Matrix Factor (MF) extracted individual blank plasma samples (20 blank plasma samples from different healthy volunteers including two lipemic and two hemolytic samples for each concentration level) spiked with standard of extraction solutions in mobile phase at the concentrations of QC1 and QC4 (after extraction) were analyzed; the peak areas were compared to the same standard of extraction solution peak areas in mobile phase. The matrix factors obtained for lisdexamfetamine were 1.458 for QC1 and 1.384 for QC4, suggesting that a significant ionization enhancement occurs in the presence of matrix ions. Meanwhile, no significant ionization suppression was observed for dexamfetamine (matrix factors were 0.986 for QC1 and 0.948 for QC4). The IS normalized matrix factors were 0.994 (QC1) and respectively 0.992 (QC4) for lisdexamfetamine/lisdexamfetamine-d4, with CVs below 1.3%, therefore adequate for reliable bio-analytical assay irrespective of the plasma properties (regular, lipemic or hemolytic). The IS normalized matrix factors were 1.049 (QC1) and respectively 1.055 (QC4) for dexamfetamine/(±)-amfetamine-d8, with CVs below 1.6% therefore adequate for reliable bio-analytical assay irrespective of the plasma properties (regular, lipemic or hemolytic).

#### Carry-Over Effect

The carry-over effect was assessed by injecting blank samples after high concentrated samples (CAL 8) in six consecutive series. The analytes blank chromatographic response was supposed to be 5 times smaller than the one given by calibrator 1 samples. The internal standards blank chromatographic response was supposed to be 20 times smaller than the one given by the previous CAL8 sample. The results showed that no signal for analytes or internal standards was detectable in blank samples injected after high concentrated samples (calibrator 8) and therefore it can be concluded that no carry-over effect was present.

#### Spiked Plasma Samples Stability

The results obtained for the tested storage conditions show that lisdexamfetamine and dexamfetamine are stable in plasma for at least the following durations: up to 6 h at room temperature (benchtop stability), up to 1 week at—5°C (autosampler stability), up to 5.5 weeks below—20°C (storage stability covering the timeframe from collection of the first sample to assay of the last sample) and up to 1 week below—70°C (transport stability fully covering the transit time from the clinic to the analytical laboratory).

#### Stability of Spiked Plasma Samples Extract

The results obtained show that lisdexamfetamine and dexamfetamine are stable in plasma extracts up to 72 h when kept at 10°C nominal (upper temperature limit acceptable for short-term refrigerated storage of processed samples that are not immediately placed in the autosampler).

#### Dilution Test

Since there is always a chance that real samples have analytes levels exceeding the maximum concentrations of the calibration curves, a past-dilution method 1/4 with blank plasma was validated. The mean dilution accuracy was within the range of variation accepted for QC samples with both compounds.

#### Stock Solutions Stability

The stock solutions of the analytes were stable up to 5.5 weeks when stored below -20°C and up to 6 h if kept at room temperature. The working solutions of internal standards mix was stable up to 20 h when kept at room temperature; therefore, the same preparation could be used within a full working day.

#### System Suitability Test Solution Stability

The suitability test solutions of the analytes and internal standards were stable up to 6 days when stored below −20°C, therefore the same preparation could be used for the full study execution.

#### Blood Sampling Tubes Validation

The risk of unreliable quantitation results was excluded for all analytes after testing the used sampling tubes pre-filled with K2EDTA as anti-coagulant.

#### Plasma Hemolyzation Impact on Accuracy of Analytes Determination

The accuracies have been calculated from the samples prepared at QC3 concentration level at three hemolyzation levels (low, middle, and high) and at QC1 concentration level (middle hemolyzation level). It was concluded that the measurement accuracy was not affected in hemolytic samples.

#### Interconversion (Conversion and/or Back-Conversion) Tests

The interconversion of lisdexamfetamine and dexamfetamine was tested in spiked plasma under storage and/or preparative conditions (6 h at room temperature, 1 week at −5°C, 5.5 weeks at −20°C, 1 week at −70°C), spiked plasma samples extracts during analyses or storage (72 h at 10°C) and in whole blood samples stored in K_2_EDTA collection tubes (3 h at room temperature). The mean results at all tested concentrations (QC1 and QC4) and for all tested conditions were within the acceptance range (±15% (85—115%) vs. nominal). Since the measurements were adequate in all tested conditions, relevant analytes interconversion can be excluded.

From the results previously reported it can be concluded that the analytical method developed for simultaneous determination of lisdexamfetamine and dexamfetamine plasma levels had adequate sensitivity, precision, accuracy, and specificity for use in clinical studies. Concentrations in validation samples were estimated on the regression curves obtained from the data of the calibration samples run in the same sequence.

#### Incurred Study Samples Re-Assay for Analytical Accuracy Evaluation

In order to test the accuracy of incurred samples, four samples for each study subject, study period and analyte (two representing the maximum lisdexamfetamine and dexamfetamine concentrations and two representing the lisdexamfetamine and dexamfetamine elimination phase) were selected for systematic incurred samples re-assay (ISR). The evaluation of the ISR accuracy has been based on the percent difference between the two sets of analyses, according to formula: percent difference = [(re-assay value—initial value)/mean value] * 100. The mean accuracy results of incurred samples re-assay were 98.71% for lisdexamfetamine and 97.85% for dexamfetamine, providing sufficient confidence that the study samples concentrations obtained were accurate.

Calculations were carried out on lisdexamfetamine (chromatographic trace m/z 264.170/83.900) peak areas normalized to the internal standard (lisdexamfetamine-d4) peak areas (chromatographic trace m/z 268.210/87.900) or dexamfetamine (chromatographic trace m/z 136.040/119.000) peak areas normalized to the internal standard [(±)-amfetamine-d8) peak areas (chromatographic trace m/z 144.094/127.100]. The calculations of concentrations were performed using weighted (1/x2) linear regression models.

There were no interferences of endogenous compounds at the retention times of lisdexamfetamine, dexamfetamine or internal standards for double blank plasma, blank plasma, samples spiked at LLOQ concentration and subject samples at C_max_ after oral administration.

### Pharmacokinetic Results

A total of 32 healthy male and female volunteers were enrolled in the bioequivalence study. All subjects were Caucasian with the mean age of 33.13 years (range 18–45 years) and mean BMI of 26.09 kg/m^2^ (range 19.0–29.8 kg/m^2^). All of the enrolled subjects received one dose of IMP during the first study period and 29 of them received also the cross-over treatment during the second period. The three subjects not completing the clinical part of the study have dropped out of their own volition due to personal reasons communicated to the investigational team. While all pharmacokinetic samples collected during the study have been analysed, the Per Protocol Population for bioequivalence assessment was comprised only of the 29 complete datasets from subjects receiving both study treatments.

**FIGURE 1:**
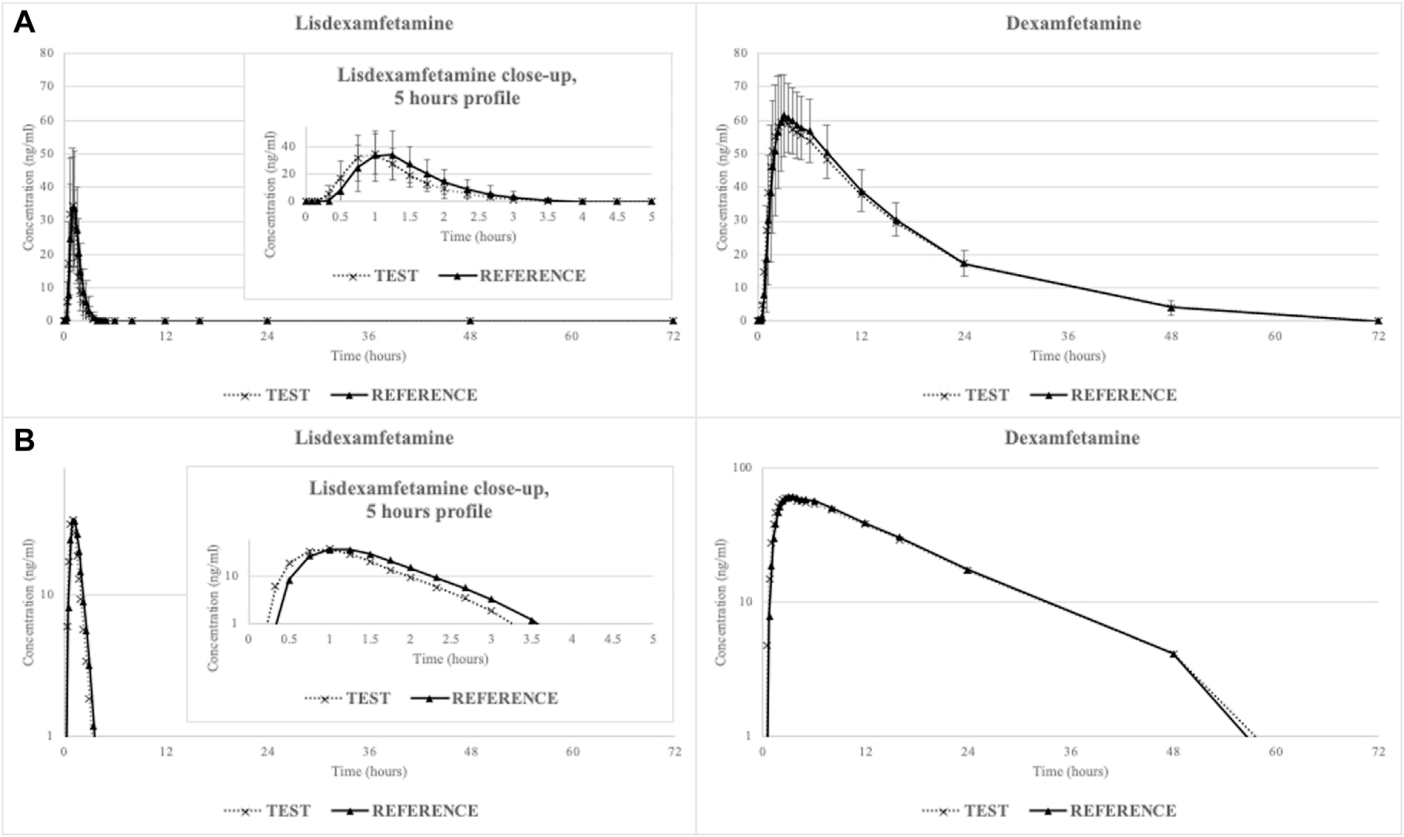
Mean lisdexamfetamine and dexamfetamine concentration-time curves (N = 29) in linear-linear display **(A)** and ln-linear display **(B)**.

The mean lisdexamfetamine and dexamfetamine concentration-time curves are shown in Figure 1 (linear-linear display in panel A and ln-linear display in panel B) while mean pharmacokinetic parameters are summarized in Table 2.

**TABLE 2:** Mean lisdexamfetamine and dexamfetamine pharmacokinetic parameters after a single oral dose of 70 mg lisdexamfetamine dimesylate consisting of either a volume of 7 ml Lisdexamfetamine dimesylate 10 mg/ml oral solution (TEST) or one hard capsule of Elvanse® (REFERENCE), administered to fasting healthy volunteers (N = 29).

PK parameter	Lisdexamfetamine		Dexamfetamine	
	Test	Reference	Test	Reference
C_max_ (ng/ml)	37.56 (36.72%)	39.62 (40.03%)	64.23 (19.76%)	64.73 (18.95%)
Mean (CV %)				
AUC_0-t_ (ng/ml*h)	42.84 (33.83%)	47.71 (37.94%)	1127.58 (17.60%)	1153.75 (18.46%)
Mean (CV %)				
AUC_0-∞_ (ng/ml*h)	43.80 (33.05%)	49.02 (36.13%)	1206.49 (18.44%)	1220.49 (19.03%)
Mean (CV %)				
T_max_ (h)	1.00 (0.75–2.00)	1.25 (0.75–2.67)	2.67 (1.50–6.00)	3.00 (2.00–6.00)
Median (range)				
MRT (h)	1.33 (24.61%)	1.66 (55.94%)	17.29 (16.74%)	17.08 (14.32%)
Mean (CV %)				
t½ (h)	0.44 (23.38%)	0.56 (127.06%)	11.42 (19.66%)	11.21 (15.68%)
Mean (CV %)				

Lisdexamfetamine was rapidly absorbed from the gastrointestinal tract, with a mean lag time of 29 ± 14 min for the reference hard capsule (requiring dissolution) and 21 ± 10 min for the test oral solution. Conversion to active dexamfetamine also occurred rapidly, with a mean lag time of 47 ± 14 min for the reference product and 35 ± 14 min for the test product.

The statistical test (Wilcoxon Signed-Rank) applied to untransformed individual T_max_ data demonstrated that there are no statistically significant differences between treatments with respect to time needed to reach maximum plasmatic concentration of lisdexamfetamine (*p* = 0.06) or dexamfetamine (*p* = 0.21).

The analysis of variance (ANOVA) ran on t_1/2_ data revealed a statistically significant difference between treatments with respect to apparent elimination half-life of lisdexamfetamine (*p* = 0.02) but not for dexamfetamine (*p* = 0.80). This statistical finding is void of any clinical significance, as it was observed for the inactive prodrug only and, furthermore, the difference between treatments in terms of mean t_1/2_ values was of only 7.2 min.

The prodrug lisdexamfetamine exhibited a short t_1/2_ (less than 1 h) and overall systemic exposure (mean plasmatic concentrations dropped below LLOQ within 4 h post-dosing), suggestive of a quick release of dexamfetamine. The t_1/2_ observed for dexamfetamine was much longer (11 h) and systemic exposure above LLOQ was maintained up to 48 h post-dosing.

The analysis of variance (ANOVA) ran on the primary pharmacokinetic parameters of the moiety considered for bioequivalence assessment (lisdexamfetamine) showed that there were no significant influences of the administration sequence, type of treatment and period of administration (all included as fixed effects in the model specifications) on C_max_ and AUC_0-t_ data (*p* > 0.05). The subject within sequence fixed effect was found to be statistically significant (*p* < 0.05) for both primary pharmacokinetic parameters. It is worth noting that this effect is almost always determined as being statistically significant in bioequivalence studies and it only indicates that the enrolled subjects have different physiological characteristics (Loprete, 2013), a desirable trait for a test group intended to be representative for the general population. The same observations were made following ANOVA evaluation of dexamfetamine C_max_ and AUC_0-t_ data.

The point estimates of lisdexamfetamine and dexamfetamine C_max_ and AUC_0-t_ pharmacokinetic ln-transformed parameters and the 90% confidence intervals for the ratios of the population means, along with the intra-subject CVs registered are shown in Table 3.

**TABLE 3:** Lisdexamfetamine and dexamfetamine point estimates (% T/R ratios), 90% confidence intervals (90% CIs) and intra-subject coefficients of variation (% ISCVs) for the primary PK parameters C_max_ and AUC_0-t_ (N = 29).

PK parameter	Lisdexamfetamine			Dexamfetamine		
	T/R ratio (%)	90% CI	ISCV (%)	T/R ratio (%)	90% CI	ISCV (%)
**C**_**max**_	96.93	88.19–106.54	21.35	99.07	96.93–101.25	4.88
**AUC**_**0-t**_	91.48	84.06–99.56	19.07	98.05	94.30–101.95	8.73

The statistical evaluation of pharmacokinetic data presented herein shows that the study formulations are bioequivalent as the test-reference ratios for the geometric means (%) of the primary PK parameters of lisdexamfetamine (C_max_ and AUC_0-t_) and their corresponding two-sided 90% CIs were contained within the predefined regulatory limits of 80.00–125.00%. Results from the statistical analysis of the additional pharmacokinetic parameter AUC_0–∞_ further support the conclusion of equivalence between the two products. The same observations can be made based on evaluation of the supportive dexamfetamine C_max_, AUC_0-t_ and AUC_0–∞_ data.

As seen with most prodrugs, the intra-subject coefficients of variation of the main disposition parameters of the parent compound were much higher than those of the released active moiety.

### Palatability Results

All of the 28 study subjects judged by the Investigator as being taste- and smell-sensitive (based on their ability to recognize the full range of tastes and odors included in the organoleptic screening) have received the test treatment and have participated in the palatability evaluation. The goal of the palatability evaluation conducted for the test oral solution was to evaluate its acceptability (projected ability to use the oral solution daily), aftertaste, bitterness, flavor, and sweetness. Each subject participated in two rounds of evaluation: the first one conducted immediately post-dose and the second one at 10 min after the administration.

**FIGURE 2:**
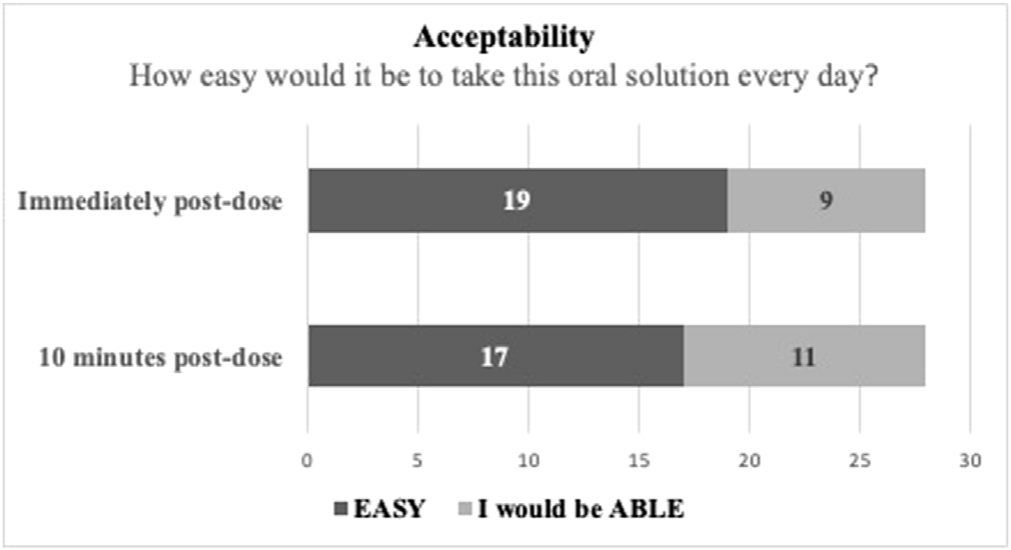
Test solution acceptability evaluation in taste- and smell-sensitive subjects (N = 28).

The results from the acceptability evaluation are presented in Figure 2, while the outcomes of the other assessments are summarized as Supplementary Material.

As observed in Figure 2, to the question “How easy would it be to take this oral solution every day?,” 19 subjects responded that it would be “easy” (67.86%) and 9 subjects responded that they would be “able” to do it (32.14%) immediately post dose. At the 10 min post-dose re-evaluation the same question produced 17 “easy” responses (60.71%) and 11 “able” responses (39.29%). These results suggest that the oral solution has a good acceptability/treatment compliance potential.

### Safety Results

A number of 21 non-serious adverse events occurred during the present study in 14 subjects. All the adverse events were deemed by the Investigator as being mild or moderate in intensity and transient. While 17 adverse events were categorized as being treatment-emergent, 4 adverse events were considered not related to the administration of the study medication (one case of menstrual pain, one case of ear pain and two cases of dental pain).

Among the treatment-emergent adverse events, the highest incidence was noted for headache (7 cases), tachycardia (5 cases) and nausea (4 cases). These adverse events do not raise any new concerns, being consistent with the known safety profile of lisdexamfetamine dimesylate (reactions listed as either very common or common in the prescribing information, based on clinical data and spontaneous reporting). There was also one isolated case of orthostatic hypotension.

The statistical analysis of adverse events (single sample proportion test applied by group of treatment) did not identify any statistically significant difference between the TEST and REFERENCE treatments with respect to the incidence of adverse events or the number of subjects having experiencing adverse events.

## Discussion

A HPLC-MS/MS analytical method for simultaneous determination of lisdexamfetamine and dexamfetamine in human plasma was developed and validated according to current regulatory requirements. The validated method was then applied for analyzing plasma samples collected during an open label, two-period, two-sequence, cross-over, randomized, single dose bioequivalence study of Lisdexamfetamine dimesylate 10 mg/ml oral solution vs. equal dose of Elvanse 70 mg hard capsule, administered in fasting conditions to 32 adult, healthy male and female volunteers.

The statistical evaluation of lisdexamfetamine pharmacokinetic data revealed that the study formulations are bioequivalent (the test-reference ratios for the geometric means (%) of the primary PK parameters C_max_ and AUC_0-t_ and their corresponding two-sided 90% CIs were contained within the predefined regulatory limits of 80.00–125.00%).

It is interesting to note that while for the lisdexamfetamine prodrug, PK results for the two formulations were slightly different due to the distinct dissolution state at administration (C_max_ and AUC_0-t_ ratios of 96.93% and respectively 91.48%), the PK parameters calculated for dexamfetamine were almost identical (C_max_ and AUC_0-t_ ratios of 99.07% and respectively 98.05%). A potential explanation of this phenomenon, already described in literature (Ermer et al., 2016), is that biotransformation of lisdexamfetamine by red blood cells (rather than its release within the gastrointestinal tract) is the process controlling the rate of dexamfetamine delivery.

Study results were checked against a published pharmacokinetic review of lisdexamfetamine (Comiran et al., 2016) and found to be similar with data obtained by other authors in a variety of distinct clinical scenarios [e.g. in elderly population (Ermer et al., 2013), in schizophrenia patients as add-on therapy to antipsychotic treatment (Martin et al., 2014), co-administered with cytochrome P450 substrates (Ermer et al., 2015)], thus reflecting the low susceptibility of the molecule’s oral bioavailability to endogenous and exogenous factors.

The observed tolerability of the study formulations was consistent with the known safety profile of lisdexamfetamine dimesylate.

The palatability evaluation conducted for the test oral solution suggests that it has a good acceptability/treatment compliance potential.

## Data Availability

The raw data supporting the conclusion of this article will be made available by the authors, without undue reservation.

## Supplementary Material

Supplementary Figure S1 Test solution palatability evaluations in taste- and smell-sensitive subjects (N = 28).
