# Supplementary figures and images for: Single Dose Comparative Bioavailability Study of Lisdexamfetamine Dimesylate as Oral Solution Versus Reference Hard Capsules in Healthy Volunteers
Source: Front Pharmacol. 2022 Apr 5;13:881198. doi: 10.3389/fphar.2022.881198 (PMC9016112; doi:10.3389/fphar.2022.881198)

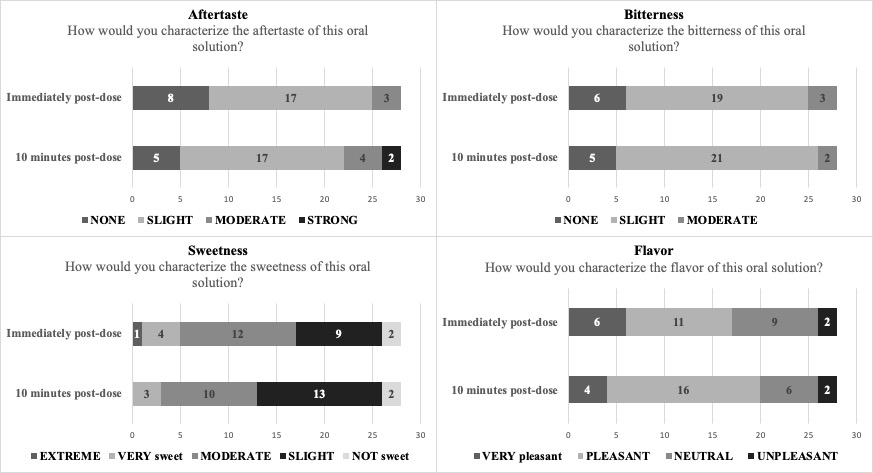

Supplement: Supplementary file 1 [file Image1.JPEG]
